# Malaria Parasite Cell Classification Using Transfer Learning with State-of-the-Art CNN Architectures

**DOI:** 10.3390/biology14121792

**Published:** 2025-12-16

**Authors:** Azhar Ali Laghari, Wazir Muhammad, Mudasar Latif Memon, Ayaz Hussain, Akash Kumar

**Affiliations:** 1 College of Resources and Environment, Shanxi Agricultural University, Taigu 030801, China; 2 Department of Electrical Engineering, Balochistan University of Engineering and Technology, Khuzdar 89100, Pakistan; engr_ayaz@yahoo.com; 3 Department of Information Technology, University of Modern Sciences, Tando Muhammad Khan 70220, Pakistan; 4 School of Civil Engineering, Guangzhou University, Guangzhou 510006, China; akash.kumar@gzhu.edu.cn

**Keywords:** medical image analysis, malaria detection, transfer learning, blood smear analysis

## Abstract

Malaria remains a critical global health challenge for doctors and healthcare practitioners, particularly clinicians involved in initial treatment. Inaccurate diagnosis of malaria-infected cells often leads to delayed or inappropriate treatment, increasing the risk of severe complications or death. Traditional microscopic diagnosis is time-consuming and requires expert skills, resulting in variability and inconsistency in results. These challenges are further complicated by the complexity of malaria symptoms, which overlap with other febrile illnesses, making clinical diagnosis unreliable without laboratory confirmation. To address these challenges, this study explores deep-learning-based approaches, particularly leveraging state-of-the-art pretrained convolutional neural network (CNN) models, for automated malaria parasite detection and classification from microscopic blood smear images. Transfer learning is an effective approach to handling issues such as limited labeled data, time-consuming training, and domain-specific variations in medical image classification. By leveraging pretrained models trained on large-scale datasets like ImageNet, transfer learning enables the reuse of learned features, significantly accelerating the adaptation process for malaria detection and other medical imaging tasks. We used eight pretrained models for malaria parasite classification such as VGG16, VGG19, Inception-v3, ResNet-18, ResNet-34, ResNet-50, ResNet-101, and Xception. In particular, ResNet-50 and ResNet-101 achieved accuracies of approximately 89%, respectively, while Xception reached around 88% accuracy. In comparison, VGG-16 achieved a lower overall accuracy of about 80% due to a recall trade-off despite high precision. These metrics highlight meaningful improvements over simpler architectures and validate the efficacy of our transfer learning approach for automated malaria detection. The proposed models were fine-tuned on extensive labeled datasets comprising parasitized and uninfected cells. Quantitative and qualitative evaluations were conducted using metrics such as precision, recall, F1-score, and support. Our experimental results demonstrate that ResNet-50, ResNet-101, and Xception exhibit strong balanced performance with higher accuracy, while VGG-16 shows a trade-off of high precision but lower recall for parasitized cells.

## Simple Summary

Malaria is a fatal disease caused by parasites transmitted via mosquito bites, and precise diagnosis is essential for efficient treatment. However, conventional diagnostic techniques, such as microscopy, are labor-intensive and require skilled staff, usually resulting in treatment delays. This article presents a technique utilizing deep learning, specifically transfer learning with cutting-edge convolutional neural networks (CNNs), to automate the detection of malaria parasites in blood smear images. The study examined eight pretrained CNN models, such as ResNet-50, ResNet-101, and Xception, attaining a maximum accuracy of 89%. The research revealed that these models, when optimized with malaria data, provide more accurate and faster diagnoses than conventional methods, rendering them particularly advantageous in resource-limited environments. The findings underscore that transfer learning can markedly decrease training duration while enhancing accuracy, presenting a valuable resource for malaria diagnosis in healthcare facilities. This automated method could significantly influence public health by improving early detection and treatment, hence aiding in the prevention of malaria transmission.

## 1. Introduction

Malaria is a severe and even fatal illness carried by parasites, the Plasmodium, which infect humans due to the bite of an infected malarial female mosquito, Anopheles. It is one of the main health issues in the world, especially in tropical and subtropical areas, where this disease has been a major source of morbidity and mortality, especially among young children and pregnant people [1,2]. Malaria remains a significant public health issue, with the World Health Organization (WHO) currently predicting 249 million cases and 608,000 deaths annually all over the world [3]. The illness can result in fever, chills, headaches, and muscular aches just to mention but a few. Unless it is detected and treated far apart, it may cause severe repercussions by resulting into severe anemia, kidney failure, cerebral malaria, and acute respiratory distress [4].

Malaria as a complex parasite has a complicated life cycle involving the host, which is human as well as mosquitoes. Among humans, parasites first attack the cells of the liver, then proliferate to attack red blood cells, which results in the typical symptoms and pathology of the disease [5]. The management of malaria is based on a multi-pronged strategy that involves control of vectors (avoiding bites by mosquitoes), early diagnosis, and prompt treatment with antimalarial therapy [6]. Early and correct diagnosis is important in handling patients well, eliminating serious disease, and control of transmission. Conventionally, the diagnosis has greatly been based on the microscopic analysis of blood smears, a very laborious, time-consuming technique which greatly relies on the proficiency and experience of the microscopist [7]. Recently, convolutional neural networks (CNNs) and other deep learning methods have provided a viable solution to the problem of identifying methods for the automated, fast, and accurate detection of malaria parasites in microscopic images [8,9]. These are sophisticated computer techniques that can be used to analyze blood smear images in high performance, helping faster diagnosis, especially in low-resource setups, and enhancing disease management and control activities [10].

The main objectives of this study are as follows:

- To demonstrate the effective use of deep learning, specifically transfer learning with eight pretrained CNN models, to automate and improve malaria parasite classification from microscopic blood smear images, addressing the challenges of traditional diagnoses.
- To show that leveraging pretrained models (e.g., ResNet-50, ResNet-101, Xception) fine-tuned on large labeled datasets accelerates training and improves classification performance despite limited labeled medical data.
- To provide quantitative and qualitative evaluation using precision, recall, F1-score, and support metrics, revealing that ResNet variants and Xception deliver balanced accuracy, while VGG-16 achieves high precision with lower recall, guiding model selection for malaria diagnosis.

The remainder of this paper is organized as follows: Section 2 reviews related work on CNN-based malaria cell detection techniques. Section 3 presents details of the state-of-the-art transfer learning models. Section 4 discusses the materials and methods. Section 5 presents the experimental results. Finally, Section 6 concludes with a summary of the findings and potential directions for future research.

## 2. Related Work

Automated malaria-diagnosis systems have been developed widely, using traditional machine learning methods [11]. These techniques are typically performed by hand, manually retrieving features of blood smear images and then classifying them using algorithms like support vector machines or random forest algorithms [12]. Though they can be effective, to an extent, traditional strategies are largely dependent on the high-quality aspects that were manually developed by professionals and are sensitive to differences in image staining and quality [13]. In addition, these approaches usually have difficulties in terms of solidness and the ability to generalize to different datasets [14].

Deep learning (DL) has been incredibly successful in the diagnosis of malaria through automation, which is greatly improved in comparison with the conventional method. Indicatively, a 19-layered convolutional neural network (CNN) was used to obtain a high level of accuracy (98.9%) in classifying infected and uninfected cells with malaria [15]. The use of multi-wavelength imaging methods has also strengthened the integrity and speed of malaria classification systems in the sense that it adds input data. A CNN structure with five convolutional layers and two fully connected layers achieved a 97% classification rate, which highlights the effectiveness of the model in the detection of malaria [16]. More complicated structures such as deep belief networks (DBNs) have been investigated as well; through trial and error with the structures of the layer and nodes, an optimal structure was determined that had an accuracy rate of 96.21% when recognizing malaria-infected cells [17]. Models of object detection like Faster R-CNN and AlexNet were used in a two-stage object detection–classification system and the accuracy was 98% [18]. Moreover, deep models with hybrid solutions substituting the final layers of the deep models with classifiers like the support vector machines (SVMs) gave positive results, with a 93.1% accuracy in detecting falciparum malaria [9].

Muhammad et al. [19] introduce IRMRIS, an MRI image super-resolution network, also based on Inception-ResNet, which substitutes the traditional bicubic interpolation with a trainable deconvolution layer to up-sample. Majidi et al. [20] compares various approaches to the classification of malaria-infected cells and demonstrates that a designed CNN has the highest accuracy of 96.15%. The model provides an effective and stable solution to automated malaria detection and this is particularly applicable in low-resource environments. The authors of [18,21] provided a comparative analysis of five CNN-based malaria-detection models on a large dataset of images taken of the microscopic blood cells. They discovered that a simpler CNN model was able to perform well, with an accuracy of over 99%, which is a better outcome than those of more complex architectures. This shows the possibility of effective, scalable, and low-cost deep learning solutions in the diagnosis of malaria.

Akkasaligar et al. introduced a CNN and VGG16-based algorithm to classify malaria cells with their NIH dataset [22]. They talked about how these models are used to extract minute image data in this approach to identify infected cells with high accuracy. Their results indicate that the CNN model is superior, and it improves the speed and accuracy of diagnosis conducted by medical professionals. The authors of [23] proposed that the VGG-19 convolutional neural network is a suitable model of classifying the Plasmodium-infected erythrocytes with the help of optical microscope images [24]. They emphasized its great accuracy, precision, and recall, showing that it can be used in the diagnosis of malaria. They suggest that VGG-19 should be used as a convenient and efficient tool in the low-complexity laboratory settings. In [25], authors explored the application of Inception-v3 architecture using various optimizers to classify images of malaria cells. They found that the greatest accuracy, 97%, was achieved by the RMSprop optimizer, which also had the lowest level of loss. The experiment proved the efficiency of such a method of precise malaria cell classification.

Zhu et al. [26] proposed an ensemble model, ROENet, which is based on ResNet and classifies malaria parasites using an extensive dataset of blood cells. Subaar et al. [27] utilized deep transfer learning models based on ResNet-18 and ResNet-34 models to identify breast cancer in mammography images. They obtained 92% and 86.7% validation accuracies with ResNet-18 and ResNet-34, respectively, on the binary classification of benign and malignant cases. The research also created a demo web application and it showed how transfer learning can be useful in helping to diagnose breast cancer early and in environments with limited resources. An example of the application of this approach in malaria diagnosis is shown in [28], where the authors present a method of diagnosing malaria based on the ResNet-50 transfer learning on a blood smear image. Their model was more accurate and robust than traditional and other deep learning models. They highlight that it can be a useful and cost-effective diagnostic instrument in resource-constrained environments.

According to Hoque et al. [29], ResNet-101 and other variants of the ResNet can be effectively used to classify malaria parasites with a vast amount of red blood cell images. Their comparative study revealed that ResNet-50 v2, achieving the best accuracy of 94.09%, appeared to be the most effective; ResNet-101 models also performed well and revealed the effectiveness of deep residual networks in the accurate diagnosis of malaria. They emphasize that the malaria-detection models based on transfer learning and cross-validation provide effective solutions to the process of automating the detection of malaria in the clinical settings [30]. Sriporn et al. [31] showed that the Xception model with the Mish activation function and the Nadam optimizer has a high level of malaria detection, achieving an accuracy of 99.28%. These approaches were found to have improved in recall, precision, and F1-score, justifying their usefulness in automated diagnosis with blood smear images. Deep learning models can be optimized and can greatly assist medical decision making because they enhance the reliability and speed of detection.

In general, malaria-detection algorithms based on deep learning have shifted towards increased accuracy, robustness, and computational efficiency, proving the suitability of the algorithm deployed in resource-constrained clinical environments where the diagnosis of malaria is critical. Not only can these advances enhance patient outcomes, they can also serve to aid large-scale efforts to control and eliminate malaria around the world.

## 3. Transfer Learning Models

Transfer learning models are a type of machine learning that involves the reuse or transfer of the learning acquired by a model on one task to a similar but distinct task. Transfer learning also uses pretrained models that have been trained on large datasets to construct and train a model more quickly, using less data and computer processing. Popular architectures that are used as pretrained models in the context of classifying malaria blood samples include VGG-16, VGG-19, Inception-v3, ResNet variants (18, 34, 50, 101), and Xception. These models have already been trained to learn to extract useful features and patterns of images, generally of large-scale datasets, such as ImageNet.

By refining or adjusting these models on blood smear images of malaria, the accuracy of classification is increased, and less labeled data are needed. It is worth noting that, in all these models, the malaria classification task is only trained using a few numbers of epochs usually 10 epochs. This is because the pretrained models have been previously trained on ImageNet, which is a large and high-quality dataset with a diversity of images. Hence, the malaria task primarily requires that the models are updated or fine-tuned without extensive training, which saves time and computational resources. It works by using the general feature extraction properties of the pretrained models to learn the problem of malaria detection fast and to minimize overfitting and enhance strength.

**Figure 1:**
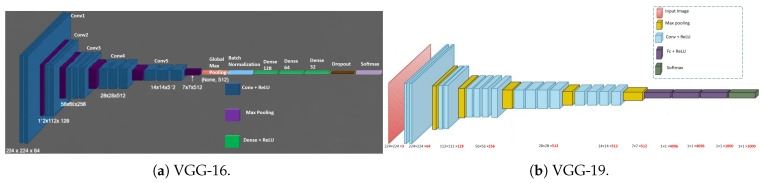

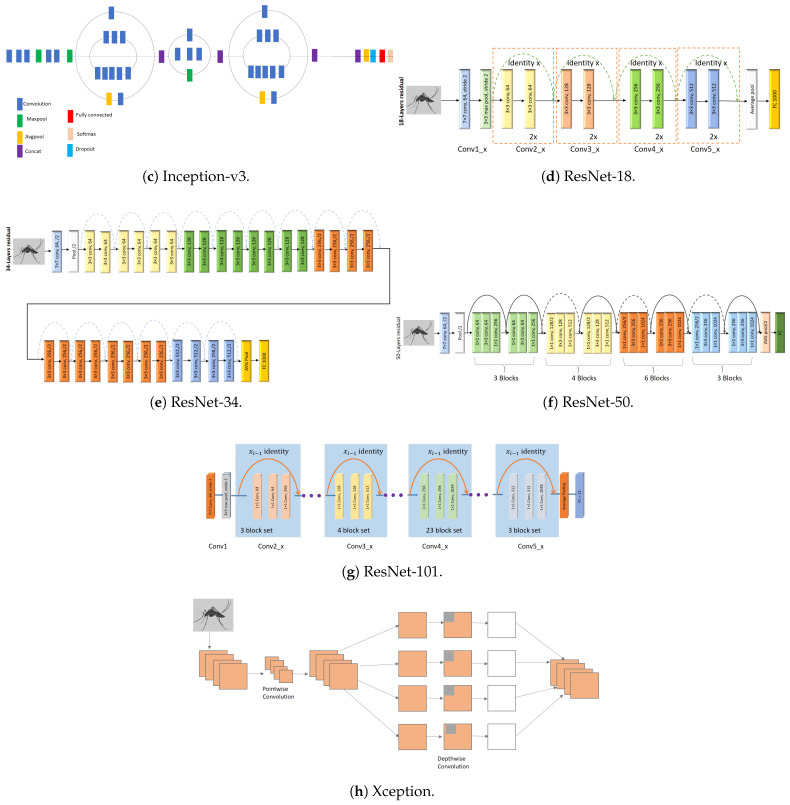
General architectures of state-of-the-art pretrained models.

Lastly, the following are succinct descriptions of each of the previously trained models that we will use in our transfer learning model of malaria blood sample classification, as illustrated in Figure 1:

**VGG-16:** It is a deep convolutional neural network that has 16 weight layers, which is composed of 13 convolutional layers and 3 fully connected layers. It also involves small 3×3 convolution filters and an ReLU activation following each layer and max pooling that reduce spatial dimensions. It was created by the Visual Geometry Group at Oxford and it has good image classification performance with a simple and homogeneous architecture. The overall structure of VGG-16 presented in Figure 1a [32,33].

**VGG-19:** VGG-19 is also a variant of VGG-16 using 19 weight layers; however, it has 16 convolutional layers with 3 fully connected layers. It shares the same architectural design as the VGG-16, and is more accurate, although at the expense of higher calculating power. The predominant structure of VGG-19 depicted in Figure 1b [33,34].

**Inception-v3:** Inception-v3 is a member of the Inception family, and the model applies an efficient architecture, which consists of convolution layers with different sizes (1×1, 3×3, 5×5) in the same module to learn the multi-scale spatial information. It is richer and more complicated than VGG models but it is configured so that it could be less costly in terms of computation. The Inception-v3 model is illustrated in Figure 1c [35,36,37].

**ResNet-18, ResNet-34, ResNet-50, and ResNet-101:** Residual networks (ResNets) solve the issue of very deep learning networks degrading by using skip connections to provide residual learning. ResNets differ in depth (18–101 layers) and are much better optimized and more accurate in that the deep layers allow easy and effortless flows in the gradient; see Figure 1d–g [38,39,40,41,42,43].

**Xception:** Xception is an extension of the Inception architecture that uses depthwise-separable convolutions, which factorize convolutions to reduce the number of parameters and maximize performance without loss of accuracy. The overall design of the Xception pretrained model is shown in Figure 1h [44,45].

## 4. Materials and Methods

### 4.1. Dataset and Data Preprocessing

This study used the NIH Malaria dataset, which is a publicly available collection of 27,558 microscopic images of red blood cells (RBCs) [46]. These images were created with the help of giemsa-stained thin blood smear slides containing samples of 150 malaria patients and 50 healthy controls giving a balanced sample of parasitized and uninfected cells. The collection contains parasitized red blood cells, which have diverse morphological changes that are characteristic of the various stages of malaria disease. Non-parasite artifacts such as dust particles or uneven staining, on the other hand, may sometimes be present in uninfected samples, which introduces actual diversity and complexity into the dataset. The dataset was split using stratified sampling, allocating 70% of images to training, 15% to validation, and 15% to testing to ensure balanced class representation. All random seeds for data splitting and training routines were fixed (seed = 42) to guarantee reproducibility. Patient-level data leakage was prevented by grouping images by patient ID, ensuring that no patient images appeared across multiple splits. The image preprocessing included resizing all images to appropriate model-specific dimensions, and normalization. Training used the Adam optimizer with a learning rate of 0.0003, batch size of 32, and early stopping based on validation accuracy with a patience of 5 epochs.

### 4.2. Hardware and Software Configuration

The experiments were conducted in the Google Cloud Platform (GCP) which consists of high-performance virtual machines with state-of-the-art GPUs and TPUs. This high-performance computing platform enabled an easy scale and greatly enhanced the data processing speed, enabling large and complicated scalable model training and evaluation procedures to be executed in a convenient time frame. The cloud environment was also flexible in providing the ability to dynamically allocate resources based on the computational requirements of various stages of the experiment that improve overall productivity.

The Python 3.8 software stack was used in the experiments, as it is widely supported in the machine learning community. TensorFlow 2.x was the major deep learning platform, which offers a comprehensive collection of neural network construction, training, and deployment tools. Some of the complementary libraries that made model development easier via high-level APIs were Keras and NumPy; Pandas were used to perform data manipulation, preprocessing, and analysis tasks effectively. This software and hardware combination made it possible to achieve a reliable, scalable, and reproducible environment necessary to perform state-of-the-art research on malaria detection using deep learning.

### 4.3. Performance Evaluation

In order to determine the performance of the model, our study evaluated the model with various evaluation metrics in order to measure the various dimensions of classification accuracy and reliability. These were precision, recall, F1-score, and overall accuracy. The model was then tested on a separate held-out dataset, after the training, to test its objective capability to correctly classify new unseen data. Moreover, a confusion matrix was used to disaggregate the results of the prediction into four categories; true positives (TPs), true negatives (TNs), false positives (FPs), and false negatives (FNs). This matrix makes it easy to specifically examine the kind of errors committed and the classes strengths of the model. Each of the measures is explained here, before reporting the findings:

**Accuracy:** Accuracy is the percentage of all the correct samples (both negative and positive) from the total predictions. It is calculated by:

(1) $$\mathrm{Accuracy}=\frac{TP+TN}{TP+TN+FP+FN}$$

**Precision:** Precision is a metric that determines the percentage of positive predictions that is correctly predicted by the model (i.e., false positives are avoided). It is given by

(2) $$\mathrm{Precision}=\frac{TP}{TP+FP}$$

**Recall:** Recall is the ratio of correct recognition of the real positives by the model, which underscores its ability to accept positive cases and not reject them. It is expressed as:

(3) $$\mathrm{Recall}=\frac{TP}{TP+FN}$$

**F1-score:** F1-score represents the harmonic mean of the accuracy and recall, integrating both the false positives and the false negatives into a single digit, which is specifically helpful when the distribution of the classes is not equal. Recall represents the percentage of the real positives that have been recognized by the model, where it is possible to identify the presence of positive cases without false alarms. It is expressed as:

(4) $$F1\text{-}\mathrm{score}=\frac{2\times (\mathrm{Precision}\times \mathrm{Recall})}{\mathrm{Precision}+\mathrm{Recall}}$$

Combined, these measures are a complete framework to assess the performance of the model in classifying it in a variety of views. It is a method of making sure that the frequency of the model being correct is tested as well as the capability of the model to deal with various prediction errors, which strengthens the reliability and the robustness of the method when applied in practice.

**Figure 2:**
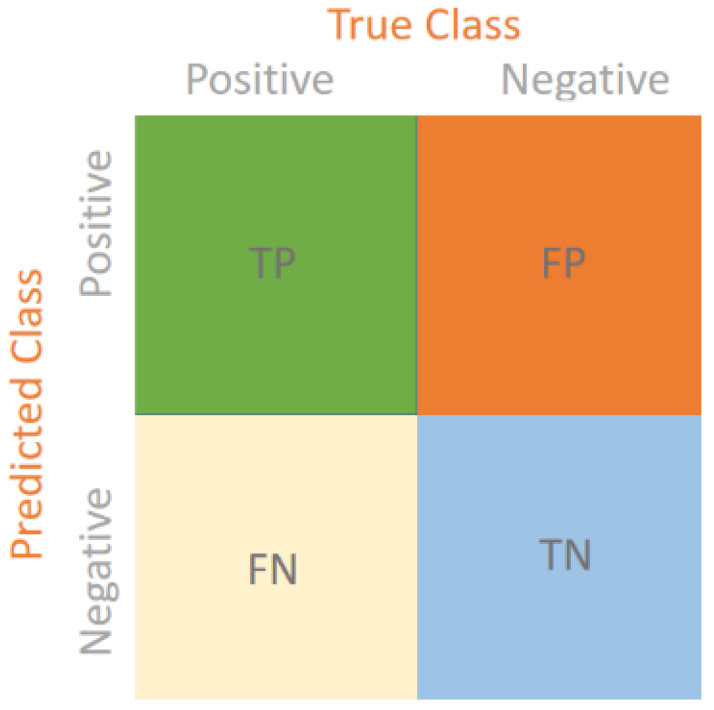
Confusion matrix.

**Confusion Matrix:** This is one of the most important instruments in machine learning because, once there is a confusion matrix, the results of the categorization algorithms are evaluated by the comparison of the predicted and the actual labels in the dataset. It is presented in the form of a table dividing the predictions into four major groups true positives (TPs), true negatives (TNs), false positives (FPs), and false negatives (FNs), as shown in Figure 2. True negatives are correctly predicted negative cases and true positives are correctly predicted positive cases. False negatives are events where the model does not identify a positive case and instead incorrectly labels it as negative (type II error), whereas false positives are the cases where the model incorrectly assigns a case as positive when it is negative (type I error).

**ROC:** The Receiver Operating Characteristic (ROC) curve is a graphical tool in the field of machine learning that is used to determine the performance of binary classification models in different threshold configurations. It plots the true positive rate (TPR), also known as the sensitivity or recall, on the y-axis vs. the false positive rate (FPR) on the x-axis at various classification thresholds. The true positive rate is the percentage of accurate positive cases that the model has identified, and the false positive rate is the percentage of negative cases that were wrongly classified (treated) as positive cases. An important summary measure that is obtained using the ROC curve is the Area Under the Curve (AUC), which quantifies the total ability of the model to discriminate between positive and negative classes. An AUC of 0.5 indicates that the performance of a model is no better than chance, and an AUC of 1 indicates that the performance is perfect.

## 5. Experimental Results

### 5.1. Training Performance Analysis of CNN Fine-Tuning

**Figure 3:**
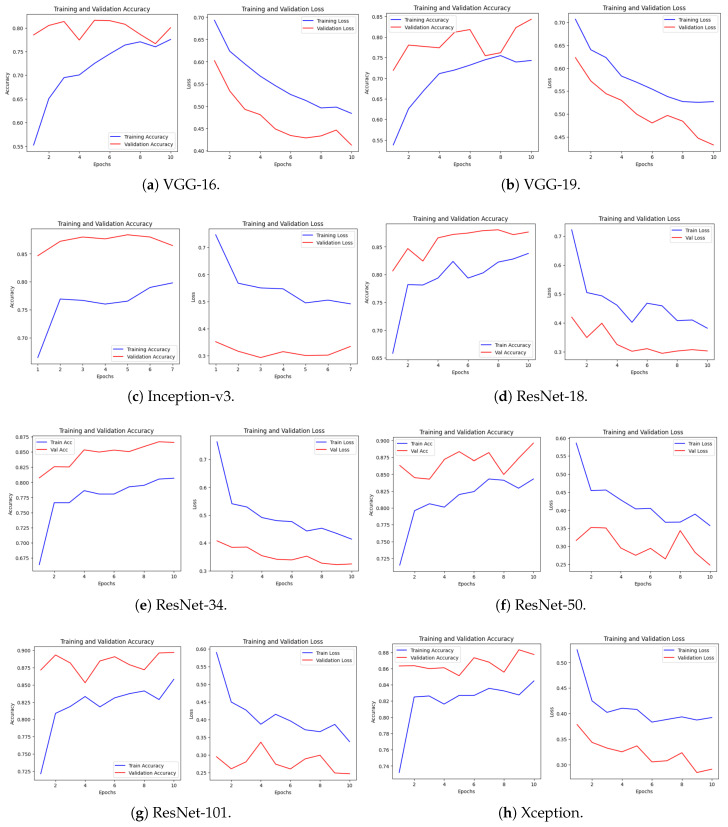
Training and validation performance of eight pretrained CNN models.

Figure 3 shows the training (blue lines) and validation (red lines) performance using both the accuracy and the loss of the architectures, e.g., VGG-16, ResNet, and Xception. All models across the board show the desired behavior of an increase in training accuracy and a decrease in training loss over the 10 epochs. However, the most important finding is the different levels of the gap that appears between the training and validation curves, which can be regarded as the important indicator of the generalization capability of the model and the possibility to cause overfitting. In such models as VGG-16 or Xception, one can see, as an example, that there is a strong distinction in the loss curves, implying that overfitting with Xception is strong early on, even though the ultimate validation accuracy is much higher with Xception.

In contrast, the ResNet family (e.g., ResNet-18 and ResNet-34) has a tendency to exhibit less aggressive overfitting, but the training and validation curves of ResNet are relatively close; however, the validation accuracy curves can be somewhat oscillatory. All these plots demonstrate the influence of various architectural options, such as the simpler VGG, and the more complicated residual (ResNet) and depth-separable (Xception) networks, on the learning dynamics and generalization performance of the specified task. It is a strategic implementation of transfer learning to decide to only perform 10 epochs of training on these models, which is notable since they are all trained on the huge ImageNet data. This period of brief training mainly aims to ensure that the new task-specific data of interest is learned, which is often smaller in size, by merely updating weights sufficiently to achieve the goal.

The models have already taken a significant amount of time computing universal image features (such as edges and textures), and these first layers are also great, stable feature extractors. To continue, hundreds of epochs would be to run the risk of universal amnesia, and the destruction of this fundamental precious knowledge. More to the point, given that fine-tuning is performed on a relatively small dataset, long training would cause the high-parameter models to memorize the noise and other anomalies of that small training set, and in fact directly result into the overfitting that is seen in the plots (increasing validation loss and decreasing training loss). As such, the few epochs are fine-tuned, i.e., the weights of the later layers are slightly adjusted with a small learning rate, which gives subtle hints to the previously learned features to ensure that they are relevant to the new task without unlearning the underlying ImageNet knowledge. The 10-epoch constraint is a critical hyperparameter selection that balances the adaptation of the existing knowledge and overfitting on the smaller target dataset effectively and the model can be quickly trained to find an optimal balance. Furthermore, NVIDIA T4 GPUs are used in our experiments; these are available on the Google Cloud Platform, which provides 8 virtual CPUs and 30 GB of RAM. The system is equipped with either an NVIDIA Tesla T4 GPU, featuring 16 GB of GDDR6 memory, or an NVIDIA V100 GPU with 32 GB of HBM2 memory, depending on availability. The utilized GPU drivers are NVIDIA CUDA version 12.x alongside cuDNN 8.x. The storage for the experiments is provided by a 200 GB SSD persistent disk. The software environment consists of Python 3.8, TensorFlow 2.10 or higher, and Keras 2.10 or above. These configurations enabled efficient training of all eight convolutional neural network models over 10 epochs on the NIH Malaria dataset, with a batch size of 32; typical training times range from approximately 2 to 4 h per model.

### 5.2. Performance Evaluation of Transfer Learning Models

The transfer learning models were critically tested on the test set of the NIH Malaria dataset, and the overall performance was quite impressive. Table 1 indicates the performance of various quality matrices (including precision, recall, F1-score, support, accuracy, macro average, and weighted average) of the findings of the confusion matrices for various pretrained models. Precision is a measure of the correct positive predictions among all that are predicted to be positive. In VGG-16, with the parasitized class, the accuracy was 0.80: here, 80 percent of images were classified as parasitized and were indeed parasitized. The proportion of true positives in relation to actual positives is the measure of recall.

In the parasitized VGG-16, recall is 0.66, i.e., the model identified 66 percent of parasitized samples. F1-score is the harmonic mean of precision and recall, giving a trade-off between the two. In the case of VGG-16, the parasitized F1-score is 0.77. The number of true samples in each class is 1378. Accuracy is the ratio of the total number of correct predictions of the two classes. Macro average simply calculates the mean measure of each of the classes, and they are considered equal. The weighted average computes the arithmetic measure, which includes the support (class imbalance). The general performance summary for VGG-16 is also precise, with high parasitization and lower recall; thus, the summary does not capture all infected cases and this affects the general accuracy (80%).

VGG-19 is more balanced with precision and recall (both approximately 0.83), and its accuracy is 83 percent. Inception-v3 is further enhanced to balanced precision and recall, achieving 0.87, with an accuracy of 87%. ResNet-18 is similar to Inception-v3 in terms of performance (87%). ResNet-34 has a smaller accuracy at 85%. Optimal accuracy (89%) and high precision and recall in both classes were achieved by ResNet-50 and ResNet-101. Xception achieves 88% accuracy and high recall when there is no infection (0.95), but recall was a bit lower when there was parasitization (0.81).

**Figure 4:**
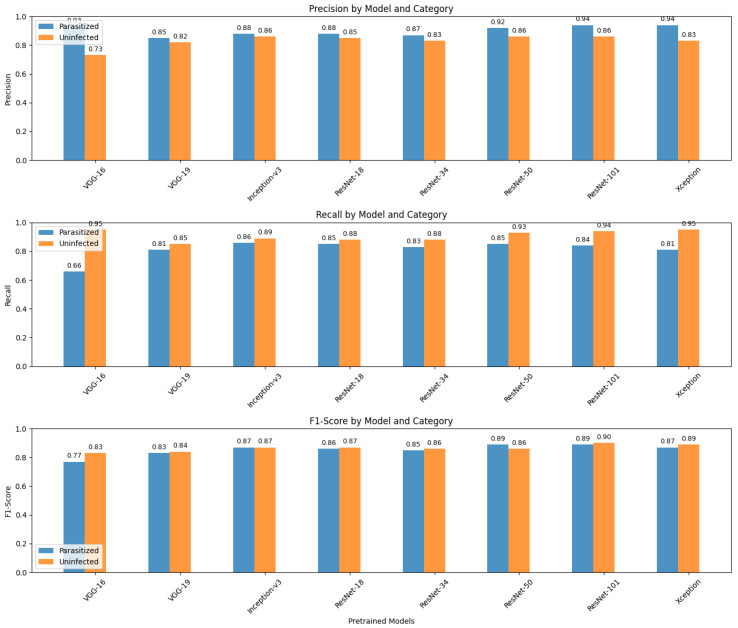
Comparison of classification metrics (precision, recall, and F1-score) by pretrained model and category.

Lastly, ResNet-50, ResNet-101, and Xception exhibit good balanced results with greater accuracy, whereas VGG-16 exhibited a trade-off between high accuracy and low recall on the parasitized data. This implies that the latter is likely to miss more infected samples, although it has a lower false positive rate. These metrics can be used to guide the selection of the preferred model in terms of the sensitivity and specificity required in each of the classes in the malaria-detection problem. Figure 4 displays a comparative analysis of three key classification metrics—precision, recall, and F1-score—for two categories (parasitized and uninfected) across multiple pretrained models (VGG-16, VGG-19, Inception-v3, ResNet-18, ResNet-34, ResNet-50, ResNet-101, and Xception).

The overall performance summary as the VGG-16 has high precision for parasitized but lower recall, so it misses some infected cases, negatively impacting the overall accuracy (80%). VGG-19 balances precision and recall better (both ∼0.83), improving accuracy to 83%. Inception-v3 improves further with balanced precision and recall of around 0.87 and an accuracy of 87%. ResNet-18 mirrors the performance of Inception-v3, also achieving 87% accuracy. ResNet-34 is slightly less accurate, at 85%. ResNet-50 and ResNet-101 have great accuracy (∼89%), with high precision and recall in both classes. Xception performs well with 88% accuracy and high recall for uninfected samples (0.95) but slightly lower recall for parasitized samples (0.81). Finally, ResNet-50, ResNet-101, and Xception show strong balanced performances with higher accuracy; meanwhile, VGG-16 shows a trade-off of high precision and lower recall for parasitized samples. This means the latter tends to miss more infected samples despite fewer false positives. These metrics can be used to inform the choice of the best model based on the desired sensitivity and specificity for each class in the malaria detection task.

### 5.3. Comparison of Pretrained Models by Total, Trainable, and Non-Trainable Parameters

**Figure 5:**
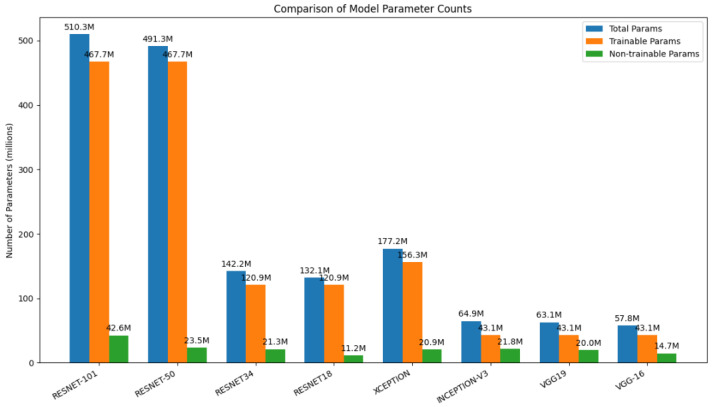
Comparison of total, trainable, and non-trainable parameters across pretrained deep learning models.

To compare the background models in terms of the number of total parameters, trainable parameters, and non-trainable parameters, Figure 5 is presented for the pretrained models. The models (ResNet-101, ResNet-50, ResNet-34, ResNet-18, Xception, Inception-v3, VGG-19, VGG-16) are plotted in the x-axis and the number of parameters (in millions) are plotted in the Y axis. The total, trainable, and non-trainable parameters have color-coded bars with approximate values marked. This plot shows that ResNet-101 and ResNet-50 have significantly more parameters than the others, with the VGG models containing the least parameters. The non-trainable parameters across the models are usually in the minority, with larger parts being trainable.

### 5.4. Memory Size Comparison of CNN Models by Total, Trainable, and Non-Trainable Parameters

**Figure 6:**
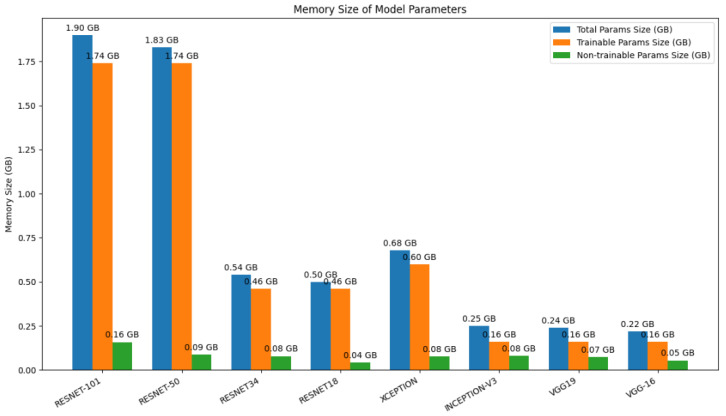
Comparison of total, trainable, and non-trainable memory sizes (in GB) for eight popular CNN architectures.

Figure 6, a bar chart, shows the memory size in Gigabytes (GBs) needed to store the parameters of eight different convolutional neural network (CNN) structures, which were split into total, trainable and non-trainable parts. The models are contrasted in terms of resource footprint, which is an important parameter to deploy. One can immediately see that the two ResNet family models, namely ResNet-101 (1.90 GB) and ResNet-50 (1.83 GB), are the most memory-sensitive, with their trainable parameters comprising a major part of the total memory. In sharp contrast, other models, like Inception-v3 (0.25 GB), VGG-19 (0.24 GB), and VGG-16 (0.22 GB), are much more memory-efficient and occupy less than a seventh of the size of the larger ResNets. The Xception model is middling, requiring 0.68 GB. The observation of this comparison is that the trade-off between the complexity of the models and resource consumption is very high and the smaller models are much better suited for environments with limited memory or computing resources.

### 5.5. Transfer Learning CNN Models Classification Performance Comparison

**Figure 7:**
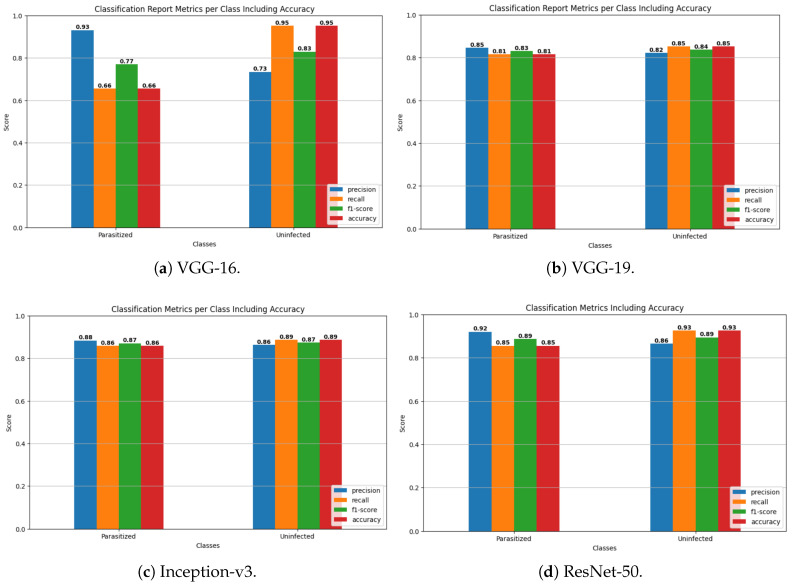

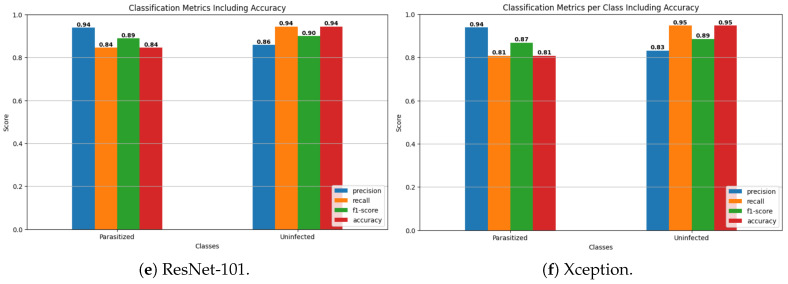
Classificationreport metrics per class including accuracy for VGG-16, VGG-19, Inception-v3, ResNet-50, ResNet-101, and Xception.

Figure 7 shows the performances of six different convolutional neural network (CNN) architectures (VGG-16, VGG-19, Inception-v3, ResNet-50, ResNet-101, and Xception) in classifying a set of two classes (parasitized, uninfected) using the performance metrics of precision, recall, F1-score, and accuracy. All the models were able to perform well (above 0.88–0.95) both classes, which means that they are powerful in terms of classification. The highest overall accuracy and balanced performance of Inception-v3 and Xception seem to be somewhat higher in comparison with the VGG models.

### 5.6. Confusion Matrices for CNN Models

**Figure 8:**
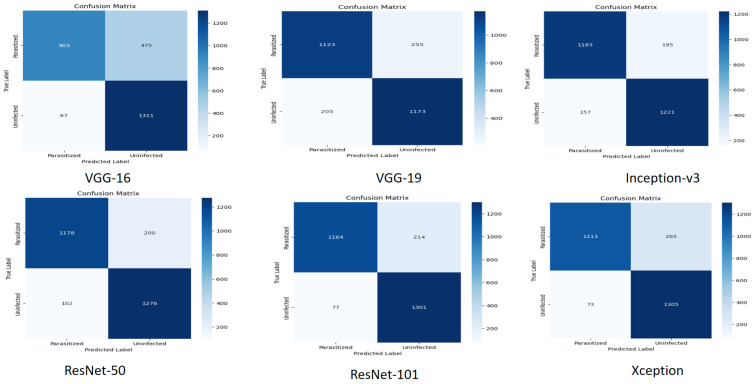
Comparison of confusion matrices across six CNN model architectures.

Figure 8 shows the confusion matrices of the six deep learning architectures (VGG-16, VGG-19, Inception-v3, ResNet-50, ResNet-101, and Xception) that categorize the cells as either parasitized or uninfected. Figure 8 provides the raw numbers of correct and incorrect predictions (true positives, false positives, false negatives, and true negatives) of six different CNN architectures. The models are shown to be mostly accurate in estimating the cases, as the diagonal values of each matrix are high. All the models display slightly elevated false negatives (a parasitized cell classified as uninfected) and false positives (an uninfected cell classified as parasitized). ResNet-101 and Inception-v3 seem to be the most balanced in terms of performance, as the numbers of correct predictions of both classes are high, and error numbers are comparatively low.

### 5.7. ROC Curve and AUC Comparison of CNN Models

**Figure 9:**
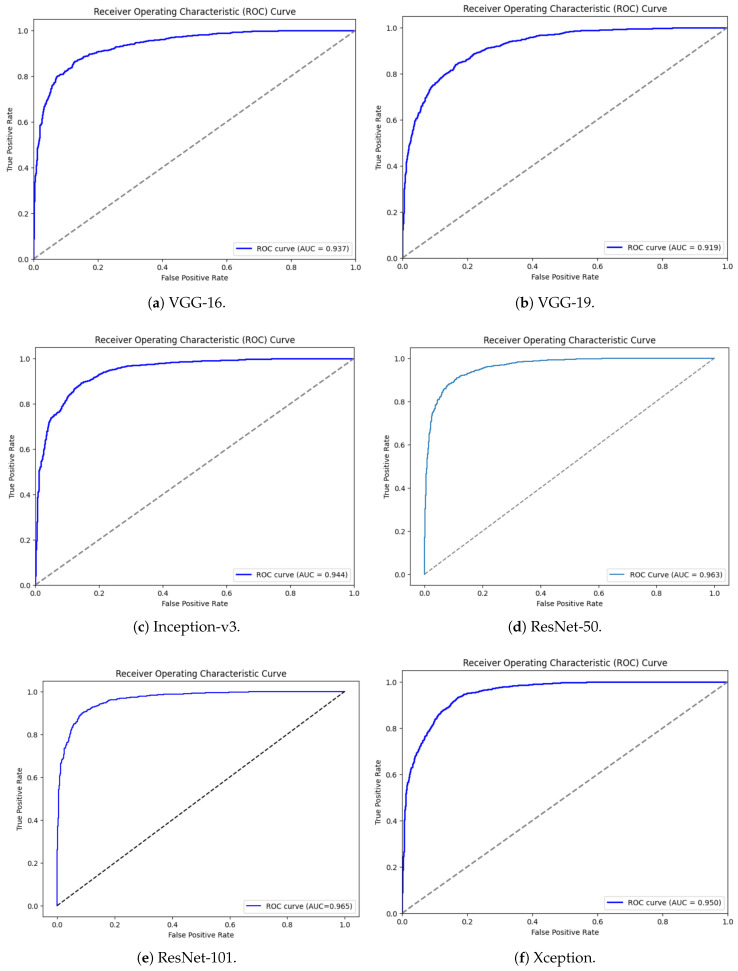
Receiver Operating Characteristic (ROC) curves with AUC values for six CNN architectures.

Figure 9 shows the Receiver Operating Characteristic (ROC) curves of the above six convolutional neural network (CNN) architectures, plotting the diagnostic capability of the architectures as the classification threshold changes. Each model has a given Area Under the Curve (AUC) with a minimum of 0.919 (VGG-19) and a maximum of 0.965 (ResNet-101). Discrimination power is excellent in all models since the ROC curves are greatly bowed up to the upper-left corner, which is far away from the diagonal line (indicating random guessing). The ResNet-family models (ResNet-50, ResNet-101) and Xception tend to have the highest AUC values, proving that they are generally more efficient in distinguishing between the parasitized and uninfected classes. In particular, ResNet-101 had the best performance with an AUC of 0.965, followed by ResNet-50 (0.963) and Xception (0.955). The high AUC values of all six deep learning models provide confirmation that they have a strong and efficient classification potential in the task at hand.

### 5.8. Prediction Counts and Classification Metrics of Transfer Learning Models

**Figure 10:**
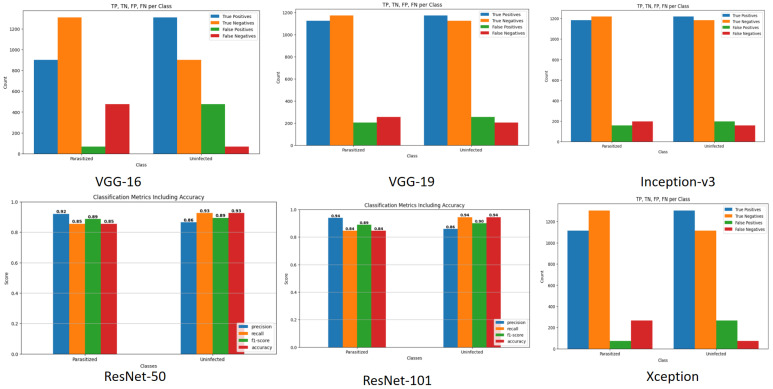
Overall view of the classification performance of TP, TN, FP, and FN counts of six models.

The top row of Figure 10 shows the prediction counts of true positives, true negatives, false positives, and false negatives for each of the six models across the “parasitized” and “uninfected” classes, visually derived from the confusion matrices. The bottom row highlights the detailed classification metrics (precision, recall, F1-score, and accuracy) specifically for the ResNet-50 and ResNet-101 architectures. The bar charts in the top row clearly show that, for all models, the number of true predictions (correctly classified samples, represented by the tall blue and orange bars) far outweighs the false predictions (misclassified samples, the shorter red and green bars). The bar charts in the bottom row demonstrate that both ResNet-50 and ResNet-101 achieved exceptional and highly balanced performance, with all key scores consistently measuring above 0.88. This overall visual evidence confirms the strong and reliable diagnostic capability of these deep learning models in accurately distinguishing between the two cell classes.

### 5.9. Qualitative Performance Evaluation of Correct and Incorrect Classifications by Transfer Learning Models

**Figure 11:**
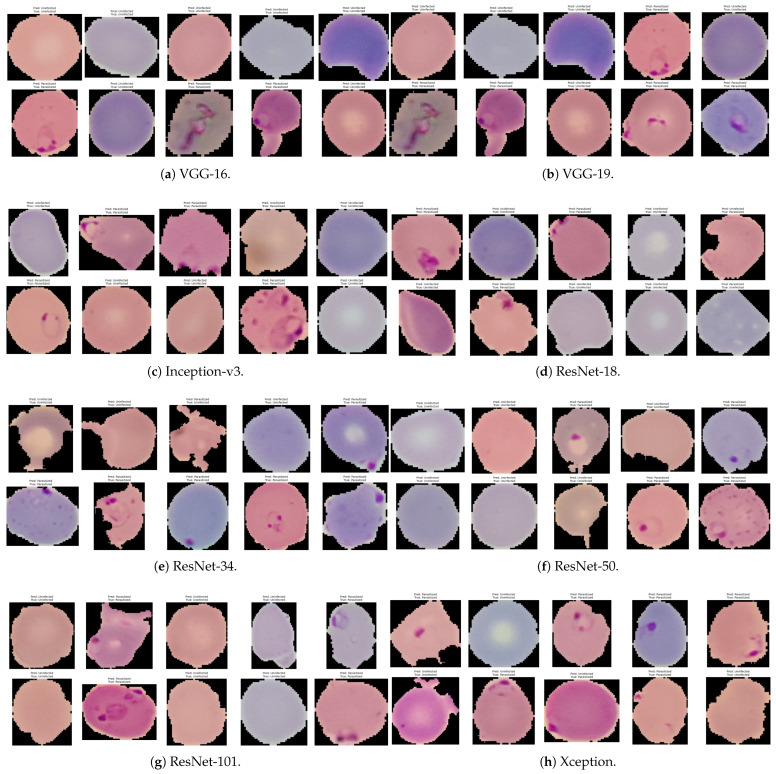
Qualitative performances of randomly selected images of eight transfer learning CNN models.

Figure 11 shows the performances of the VGG-16, VGG-19, Inception-v3, ResNet-18, ResNet-34, ResNet-50, ResNet-101, and Xception models in single-cell image classification. It presents an example of correctly matched images (where predicted label corresponds to true label) and wrongly matched images (where predicted label does not correspond to true label). It aims to give a qualitative picture of the models’ performances and to show instances in which classification can be hard because of variations in the images or low-contrast features.

### 5.10. Limitations

This study on malaria parasite classification using deep learning and transfer learning demonstrates promising results but also faces several intrinsic limitations. First, the dataset used, the NIH Malaria dataset, while publicly available and balanced, contains images from a limited number of patients and geographic regions, restricting the model’s generalizability to diverse populations and parasite strains globally. The real-world blood smear images exhibit greater heterogeneity in staining techniques, image quality, and artifacts that could impact model robustness. Second, although extensive data augmentation and preprocessing techniques were applied to mitigate overfitting and simulate variability, the models still require validation on external clinical datasets to establish their true clinical utility. The limited size of high-quality labeled datasets hinders our ability to fully capture all the biological and technical variations encountered in practice. Third, the computational resource requirements for training more complex models such as ResNet-101 and Xception remain high, potentially limiting deployment in resource-limited settings or real-time applications without further model optimization or pruning. Fourth, the study focuses primarily on binary classification of parasitized vs. uninfected red blood cells without species-level differentiation or stage classification, which are critical for appropriate therapeutic decisions. Finally, integration with other clinical and diagnostic information—such as patient symptoms, laboratory markers, and epidemiological data—could enhance predictive accuracy, but was beyond this work’s scope. Addressing these limitations in future research through the inclusion of larger and more diverse datasets, multi-class modeling, lightweight model development, real-world clinical testing, and multimodal data integration will be key to translating deep-learning-based malaria-diagnosis tools into impactful healthcare solutions.

## 6. Conclusions

Malaria diagnosis using deep learning models, particularly transfer learning with pretrained CNN architectures, demonstrates substantial progress compared to traditional microscopy techniques. The results of the present study demonstrate that transfer learning with pretrained CNN architectures offers substantial progress for automated malaria parasite detection compared to traditional microscopy. Among the eight evaluated models, ResNet-101 emerged as the clear overall leader, with 89% accuracy, and a balanced F1-score of 0.88, establishing it as the top performer for comprehensive diagnostic capability. ResNet-50 provides an optimal balance of high accuracy (88%) and computational efficiency (0.9 GB memory usage), while Xception excels in precision (0.92) for applications prioritizing minimized false positives. These results systematically reveal trade-offs between model complexity, memory requirements, and performance, confirming transfer learning’s effectiveness in overcoming limited labeled data challenges while reducing training time. However, all findings are derived exclusively from the NIH Malaria dataset without external clinical validation, limiting immediate clinical translation due to potential variations in the staining protocols, image quality, and parasite morphologies encountered in real-world settings. Future research should prioritize external validation on diverse clinical datasets, development of lightweight architectures for resource-constrained environments, and integration with multimodal diagnostic data. This comprehensive benchmark provides clear guidance for model selection while outlining essential paths toward practical clinical deployment.

## Figures and Tables

**Table 1:** Quantitative performance on different pretrained models.

Model	Category	Precision	Recall	F1-Score	Support
	Parasitized	0.93	0.66	0.77	1378
	Uninfected	0.73	**0.95**	0.83	1378
VGG-16	Accuracy			0.80	2756
	Macro Average	0.83	0.80	0.80	2756
	Weighted Average	0.83	0.80	0.80	2756
	Parasitized	0.85	0.81	0.83	1378
	Uninfected	0.82	0.85	0.84	1378
VGG-19	Accuracy			0.83	2756
	Macro Average	0.83	0.83	0.83	2756
	Weighted Average	0.83	0.83	0.83	2756
	Parasitized	0.88	**0.86**	0.87	1378
	Uninfected	**0.86**	0.89	0.87	1378
Inception-v3	Accuracy			0.87	2756
	Macro Average	0.87	0.87	0.87	2756
	Weighted Average	0.87	0.87	0.87	2756
	Parasitized	0.88	0.85	0.86	1378
	Uninfected	0.85	0.88	0.87	1378
ResNet-18	Accuracy			0.87	2756
	Macro Average	0.87	0.87	0.87	2756
	Weighted Average	0.87	0.87	0.87	2756
	Parasitized	0.87	0.83	0.85	1378
	Uninfected	0.83	0.88	0.86	1378
ResNet-34	Accuracy			0.85	2756
	Macro Average	0.85	0.85	0.85	2756
	Weighted Average	0.85	0.85	0.85	2756
	Parasitized	0.92	0.85	**0.89**	1378
	Uninfected	**0.86**	0.93	0.86	1378
ResNet-50	Accuracy			**0.89**	2756
	Macro Average	0.89	**0.89**	**0.89**	2756
	Weighted Average	0.89	**0.89**	**0.89**	2756
	Parasitized	**0.94**	0.84	**0.89**	1378
	Uninfected	**0.86**	0.94	**0.90**	1378
ResNet-101	Accuracy			**0.89**	2756
	Macro Average	**0.90**	**0.89**	**0.89**	2756
	Weighted Average	**0.90**	**0.89**	**0.89**	2756
	Parasitized	**0.94**	0.81	0.87	1378
	Uninfected	0.83	**0.95**	0.89	1378
Xception	Accuracy			0.88	2756
	Macro Average	0.88	0.88	0.88	2756
	Weighted Average	0.88	0.88	0.88	2756

## Data Availability

The data presented in this study are openly available in NIH Malaria Dataset.
